# Research progress on multiple cell death pathways of podocytes in diabetic kidney disease

**DOI:** 10.1186/s10020-023-00732-4

**Published:** 2023-10-12

**Authors:** Can Yang, Zhen Zhang, Jieting Liu, Peijian Chen, Jialing Li, Haiying Shu, Yanhui Chu, Luxin Li

**Affiliations:** 1 Heilongjiang Key Laboratory of Anti-Fibrosis Biotherapy, Mudanjiang Medical University, Mudanjiang, 157000 China; 2 College of Life Sciences, Mudanjiang Medical University, Mudanjiang, 157000 China; 3 School of First Clinical Medical College, Mudanjiang Medical University, Mudanjiang, 157000 China

**Keywords:** Diabetic kidney disease, Podocyte, Apoptosis, Autophagy, Endoplasmic reticulum stress, Pyroptosis, Necroptosis, Ferroptosis, Mitotic catastrophe

## Abstract

Diabetic kidney disease (DKD) is the main cause of end-stage renal disease, and its clinical manifestations are progressive proteinuria, decreased glomerular filtration rate, and renal failure. The injury and death of glomerular podocytes are the keys to DKD. Currently, a variety of cell death modes have been identified in podocytes, including apoptosis, autophagy, endoplasmic reticulum (ER) stress, pyroptosis, necroptosis, ferroptosis, mitotic catastrophe, etc. The signaling pathways leading to these cell death processes are interconnected and can be activated simultaneously or in parallel. They are essential for cell survival and death that determine the fate of cells. With the deepening of the research on the mechanism of cell death, more and more researchers have devoted their attention to the underlying pathologic research and the drug therapy research of DKD. In this paper, we discussed the podocyte physiologic role and DKD processes. We also provide an overview of the types and specific mechanisms involved in each type of cell death in DKD, as well as related targeted therapy methods and drugs are reviewed. In the last part we discuss the complexity and potential crosstalk between various modes of cell death, which will help improve the understanding of podocyte death and lay a foundation for new and ideal targeted therapy strategies for DKD treatment in the future.

## Introduction

Diabetic kidney disease (DKD) is caused by kidney damage due to the microvascular complications of diabetes and is the most common complication of type 2 diabetes (Long and Dagogo-Jack 2011; Jung and Yoo 2022). It is the leading cause of end-stage renal disease worldwide and is characterized by its high morbidity and mortality (Steiner 2016). It occurs in about 40% of people with diabetes (Alicic et al. 2017). The clinical manifestations are proteinuria, increased creatinine levels, and abnormal glomerular filtration rate, and end-stage renal disease develops several years later (Brosius et al. 2016; Zhou et al. 2019; Denhez et al. 2015; Manda et al. 2015). The main pathological manifestations are podocyte loss and the disappearance of the foot process, glomerular sclerosis, thickening of glomerular basement membrane (GBM), mesangial matrix expansion, interstitial fibrosis, and tubular atrophy (Manda et al. 2015; Li et al. 2007; Tuttle et al. 2022). Among these, the injury and loss of podocytes is an important early pathological marker of DKD, which can accelerate the development of DKD (Wang et al. 2014; Tunçdemir and Oztürk 2011; Benzing and Salant 2021). Podocytes are terminal differentiated epithelial cells with primary, secondary, and tertiary processes connected by a structure called the slit-diaphragm (SD), which is the only cell–cell connection structure between podocytes (Ronco 2007; Shankland et al. 2021). SD determines the filtration rate of the glomeruli, allowing free filtration of water and small molecules, but having a selective filtration effect on large molecules. The surface of podocytes is divided into two parts: the apical membrane and the basal membrane, which are located above and below the SD respectively. Integrin mediates the anchoring of podocytes basal membrane and GBM (Takeda et al. 2000).The types of podocyte death in renal diseases include apoptosis, autophagy, ER stress, pyroptosis, necroptosis, ferroptosis, mitotic catastrophe, etc. (Altintas and Reiser 2019). Each cell death type has its own unique morphological characteristics (Table 1). Apoptosis is the most common mode of podocyte death. Although autophagy is a protective mechanism for cells, it can also lead to the damage and loss of podocytes in some respects. ER stress is caused by excessively prolonged unfolded protein response (UPR) UPR. In addition, pyroptosis and necroptosis can also lead to the lytic death of podocytes. The accumulation of reactive oxygen species (ROS) during ferroptosis causes oxidative stress damage to podocytes. In mitosis catastrophe, podocytes have an inherent barrier to mitosis, which eventually leads to cell loss. Understanding the types and detailed mechanisms of podocyte death is helpful to propose novel and ideal DKD-targeted therapeutic strategies.

**Table 1:** Comparisons among various types of cell death

Cell death type	Apoptosis	Autophagy	ER stress	Pyroptosis	Necroptosis	Ferroptosis	Mitotic catastrophe
Inducement	Gene regulation under physiological conditions	Nutritional deficiency or hormone induction	Pathological stimulation	Pathological stimulation	Pathological changes or severe injuries	Pathological stimulation	Pathological stimulation
Cellular morphology	Shrinkage	Produce cavitation	The cells were enlarged and deformed	The cells were enlarged and deformed	The cells were enlarged and deformed	The cells were enlarged and deformed	The cells were enlarged and deformed
Cytomembrane	Membrane structure intact	Membrane structure intact	Membrane structure intact	Membranolysis	Membranolysis	Membrane structure intact	Membrane structure intact
Organelle	Integrity	It is phagocytosed by autophagosomes and eventually digested by lysosomes	Deformation or swelling	Deformation	Deformation or swelling	Deformation or swelling	Deformation or swelling
DNA	Degraded to fragments of 180 to 200bp and their integer multiples	Random degradation	Random degradation	Random degradation	Random degradation	Random degradation	Random degradation
References	Erekat (2022a, b, c)	Meng et al. (2019); Tang et al. (2019); Liu et al. (2022a, b)	Fan et al. (2017a, 2017b)	Cao et al. (2022)	Liu et al. (2018)	Dixon et al. (2012); Yang and Stockwell (2008)	Castedo et al. (2004); Vitale et al. (2011)

## Pathological changes of podocytes in DKD

**Fig. 1:**
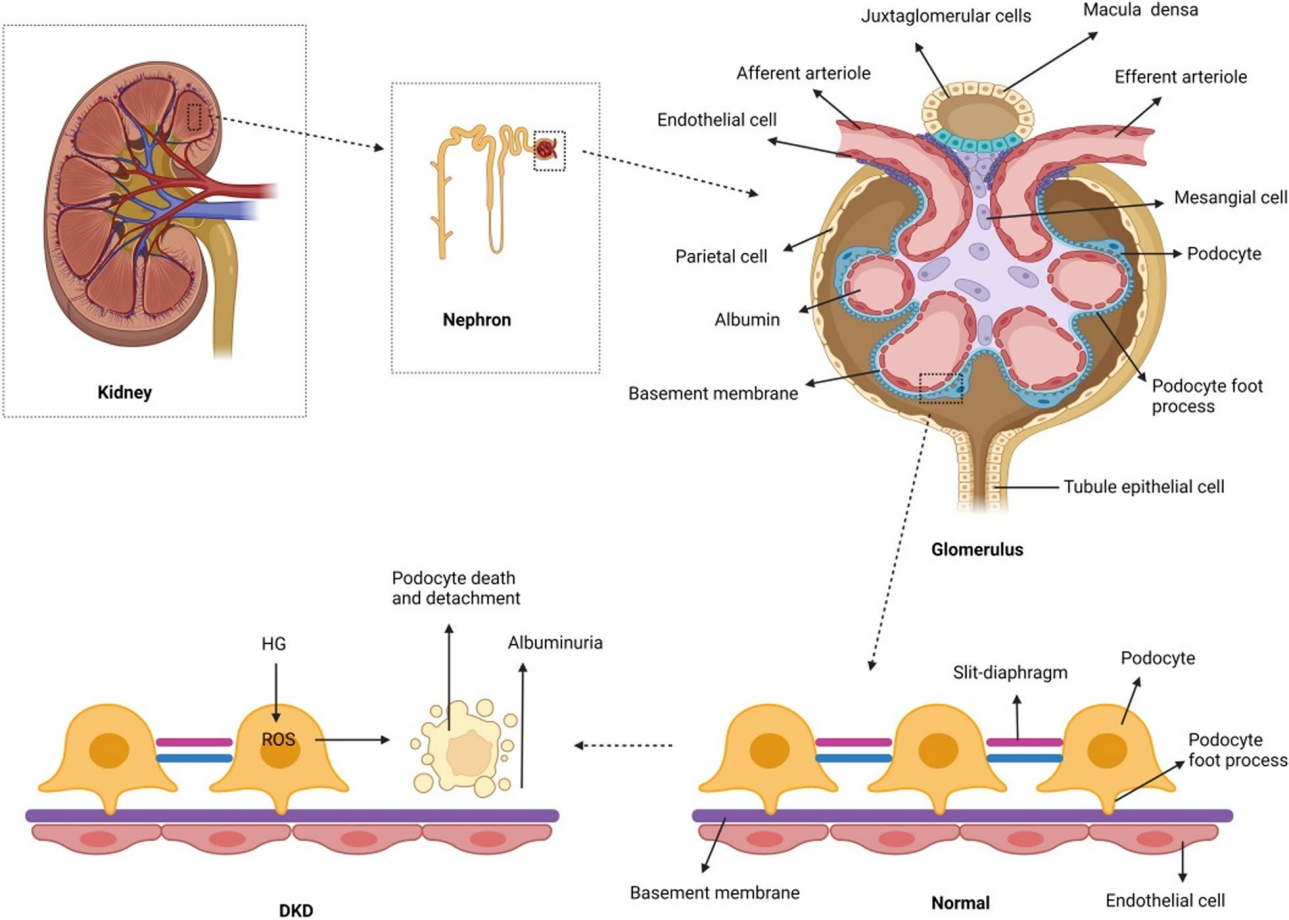
Pathological changes of podocytes in DKD. The kidney is comprised of functional units, nephrons, each of which is made of a glomerulus and a tubule. The normal healthy glomerulus includes afferent arterioles, efferent arterioles, capillary loops, endothelial cells, basement membrane, podocytes, parietal cells, and tubule epithelial cells. Foot processes from neighboring podocytes interdigitate and are connected by a modified adherent junction called a slit-diaphragm that provides intercellular space for the passage of glomerular filtrate. Podocyte foot processes, basement membrane, and endothelial cells form a tight filtration barrier in the glomerulus. Podocytes are lost due to death and detachment. Hyperglycemia-induced ROS release plays an important role in the process

The primary function of the kidneys is to maintain water, electrolytes, and acid–base balance. There are about 1 million nephrons in each kidney. The nephron consists of glomeruli and tubules. The glomeruli are responsible for filtering water and small molecules from circulating plasma, and the renal tubule system regulates their selective reabsorption and secretion, thus determining the final composition of urine (American Diabetes 2005). The filtration barrier of the glomerulus is composed of podocytes, endothelial cells, and the GBM. Among these, podocytes are characteristic end-differentiated visceral epithelial cells in the kidney, which are composed of cell bodies and primary and secondary podocytes (Podgórski et al. 2019). Podocytes adhere to the GBM mainly through α3β1 integrin (Mathew et al. 2012). The foot process of podocytes encloses the glomerular capillaries (Denhez et al. 2015). The space between adjacent podocytes is covered by the hiatus membrane, which plays an important role in glomerular filtration (Li et al. 2007; Pavenstädt et al. 2003; Chen et al. 2020). The repair and regeneration capacity of podocytes is limited in the diabetic environment due to factors, such as high glucose (HG), growth factors, fatty acids, angiotensin II (Ang II), transforming growth factor-β (TGF-β), hormones, and mechanical stretching (Liu et al. 2017; Anil Kumar et al. 2014; Wolf et al. 2005). Early renal changes in diabetes include glomerular hyperfiltration, renal hypertrophy, and microproteinuria. With the progression of DKD, the glomerular filtration rate is significantly reduced and proteinuria occurs, which eventually leads to end-stage renal disease (Burrows et al. 2014; Barutta et al. 2022). Hyperglycemia induces the production of ROS, which causes the disappearance of podocytes' foot process and the detachment or death of podocytes from the GBM, which damages the filtration barrier of the glomeruli and eventually leads to the production of proteinuria (Lin and Susztak 2016; Susztak et al. 2006; Moreno et al. 2008). The loss and death of podocytes further increase the permeability of the glomerular filtration barrier to plasma proteins, which aggravates proteinuria and leads to a vicious cycle (Castrop and Schießl 2017). Therefore, the injury and death of glomerular podocytes are crucial to the occurrence and development of DKD (Fig. 1).

## Types and mechanisms of podocyte death in diabetic kidney disease

### Apoptosis

**Fig. 2:**
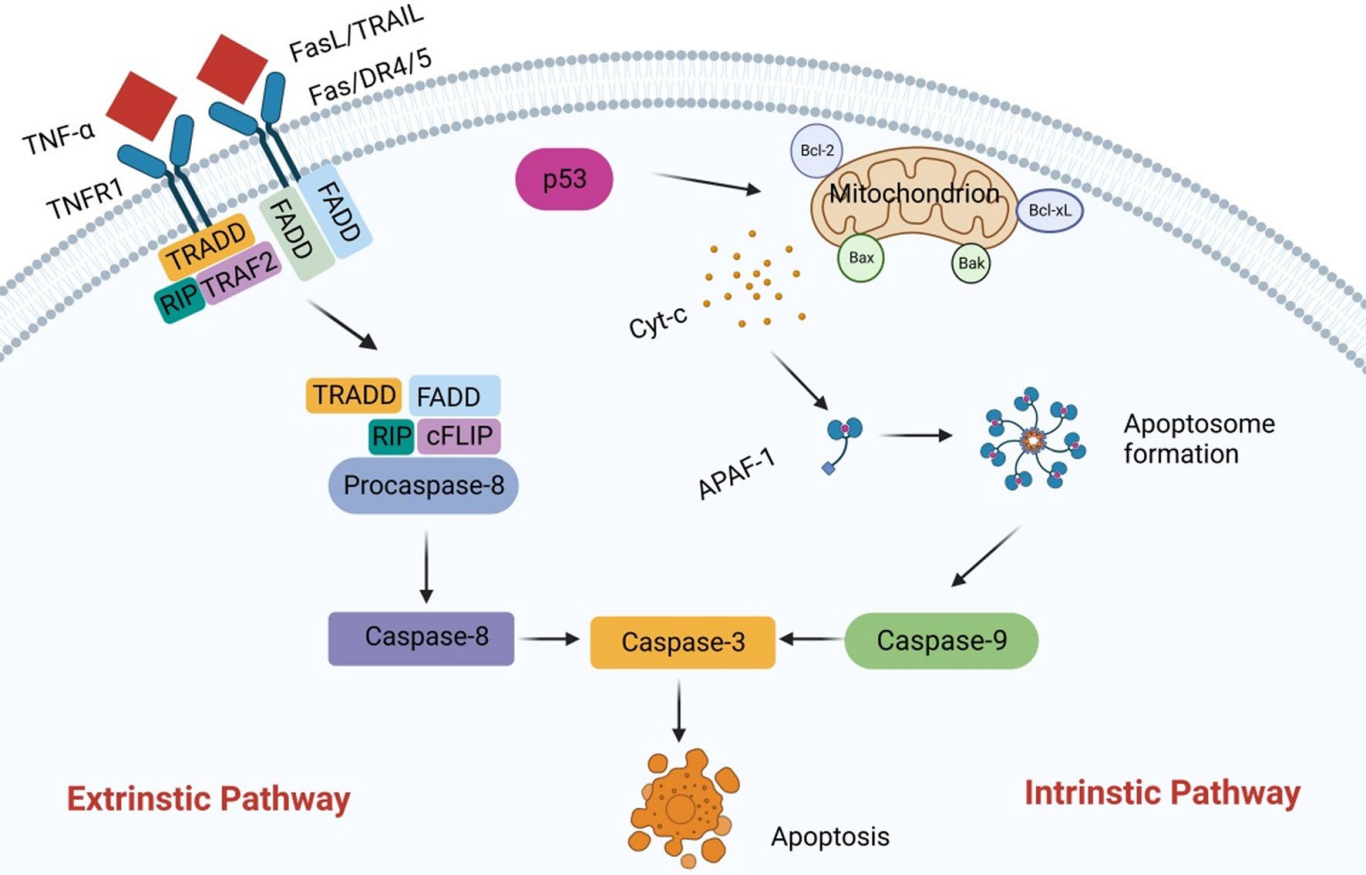
Apoptosis-related pathway. There are two main ways of apoptosis. In the extrinsic pathway, the death receptor binds to its corresponding ligands, including Fas, TNFR1, DR4, and DR5, and the corresponding ligands include FasL, TNF-α, and TNF-associated apoptosis-inducing ligand (TRAIL), which activate caspase 8 and subsequently caspase 3. It leads to apoptosis. In the intrinstic pathway, when DNA is damaged, the pro-apoptotic proteins Bax and Bak are activated, and the anti-apoptotic proteins Bcl-2 and Bcl-xL are inhibited. Subsequently, a series of apoptotic factors are released, including cytochrome c, APAF-1, and procaspase 9, which form a complex called the apoptosome. This complex can activate caspase 9, which in turn activates caspase 3, and ultimately leads to apoptosis

Apoptosis is a form of programmed cell death. The most important feature of which is the proteolytic cascade induced by caspases. Caspases exist widely in cells in the form of an inactive zymogen. When they are activated, other procaspases immediately start protease cascade reactions according to their proteolytic activity. This proteolytic cascade amplifies the apoptotic pathway, ultimately leading to rapid and irreversible cell death (D'Arcy 2019). The morphological changes of apoptosis include cell shrinkage, chromatin agglutination, DNA fragmentation, and the formation of apoptotic bodies, which are eventually cleared by phagocytosis to prevent them from causing any inflammation (Erekat 2022a, b; Erekat 2017; Erekat 2022a, b). There are two distinct pathways of apoptosis, namely the extrinsic pathway and intrinstic pathway (Fig. 2) (Tummers and Green 2017). The extrinsic pathway is also called the death receptor pathway. The intrinstic pathway is also known as the mitochondrial pathway (D'Arcy 2019; Li et al. 1997; Du et al. 2000; Goldstein et al. 2005; Kim et al. 2006; Youle and Strasser 2008). The Mitogen-activated protein kinase (MAPK) pathway plays an important role in regulating apoptosis. MAPK is a serine/threonine protein kinase that can be activated by extracellular stimuli, including cytokines, cellular stress, hormones, and neurotransmitters. The MAPK signaling pathway regulates a variety of biological processes through a variety of cellular mechanisms. In the process of apoptosis, MAPK has a dual role, it can act as either an activator or an inhibitor, depending on the cell type and associated stimulus. The MAPK signaling pathway is mainly composed of p38MAPK, C-Jun N-terminal kinase (JNK) and extracellular regulated kinase 1/2 (ERK1/2). Relevant studies have found that the activation of JNK and p38MAPK can promote apoptosis, while the activation of ERK1/2 can inhibit apoptosis. Therefore, MAPK signaling pathway has certain specificity in the process of apoptosis (Yue and López 2020).

HG is associated with the pathogenesis of DKD. Whether in vivo or in vitro, HG can induce the production of ROS, which triggers podocyte apoptosis and subsequent podocyte decline, which leads to a decrease in podocyte numbers and glomerular damage, ultimately leading to the development of DKD (Chen et al. 2015). Therefore, podocyte apoptosis and subsequent podocyte decline may influence the early events of diabetic kidney and potential diabetic glomerulopathy, leading to DKD in both type I and type II diabetes (Wang et al. 2021; Eisenreich and Leppert 2017). Therefore, the production of excess ROS may be one of the mechanisms of the occurrence of DKD (Rask-Madsen and King 2013; Nishikawa et al. 2000; Giacco and Brownlee 2010). Thioredoxin interacting protein (TXNIP), also known as vitamin D-upregulated protein 1 (VDUP-1) or thioredoxin binding protein-2 (TBP-2), has been reported to play an important role in the regulation of ROS (Lu and Holmgren 2014). Further exploration of its mechanism shows that TXNIP, as an endogenous thioredoxin (Trx) inhibitor, binds to the oxidation-reducing cysteine residue in thioredoxin, thus inactivating the antioxidant function of thioredoxin and becoming a key component of cellular REDOX regulation (Nishiyama et al. 1999). Shah et al. experimentally demonstrated that TXNIP deficiency inhibits diabetes-induced cell surface matrix accumulation, renal fibrosis, podocyte deletion, and podocyte process disappearance. In HG-cultured podocytes, knocking down the TXNIP gene by siRNA terminates mitochondrial superoxide (O^2−^) production, thereby reducing podocyte apoptosis (Shah et al. 2015). Under pathological conditions, the overproduction of ROS activates the antioxidant defense system, resulting in cellular oxidative stress, damage to cellular oxidative components, and ultimately the regulation of cell apoptosis. In contrast, targeting cellular oxidative stress-related pathways protects podocytes from apoptosis (Zhu et al. 2022; Yang et al. 2016). For example, The overexpression of Sestrin2 alleviates oxidative stress by coordinating the TSP-1/TGF-β1/Smad3 pathway and alleviating apoptosis and injury to podocytes in DKD (Song et al. 2022). In addition to the important influence of oxidative stress on the pathogenesis of DKD, the abnormal regulation of microRNAs (miRNAs) has also been confirmed to be related to the occurrence of DKD (Simpson et al. 2016). miRNAs are single-stranded non-coding RNAs with a length of 19–22 nucleotides that are important regulators of post-transcriptional gene expression and play key roles in physiological and pathological processes, such as cell survival, proliferation, differentiation, apoptosis, and the immune response (Mendell and Olson 2012). For example, exosomes secreted from adipose-derived stem cells (ADSC-Exo) increase the expression of miR-486 in podocytes, thereby inhibiting the Smad1/mTOR signaling pathway and reducing podocyte apoptosis (Jin et al. 2019).

As for the targeted treatment of DKD podococyte apoptosis, in addition to the related pathological studies, there are also some drug studies. For example, astragaloside IV (AS-IV) also can reduces podocyte apoptosis by activating the PPARγ-Klotho-FoxO1 signaling pathway to inhibit oxidative stress, thereby improving DKD (Xing et al. 2021). In addition, swiprosin-1 is a protein that mediates HG-induced podocyte apoptosis and plays an important role in the development of DKD but can be treated by telmisartan, which mainly ameliorates HG-stimulated mitochondria-dependent podocyte apoptosis through the p38 MAPK signaling pathway (Wei et al. 2022). The Bcl-2 gene has an obvious inhibitory effect on apoptosis and is one of the oncogenes that has received much attention recently. Liu and his team evaluated the in vivo and in vitro effects of wogonin on DKD podocytes using HG-induced MPC5 cells and streptozotocin (STZ)-induced diabetic mouse models and found that wogonin enhanced the activation of anti-apoptotic Bcl-2. It also alleviated the podocyte apoptosis mediated by Bax in DKD, and thus, it is expected to be a promising drug for the treatment of DKD (Liu et al. 2022a, b). In summary, the above events suggest that compounds or molecules that inhibit apoptosis can be used as potential therapeutic agents for DKD.

### Autophagy

**Fig. 3:**
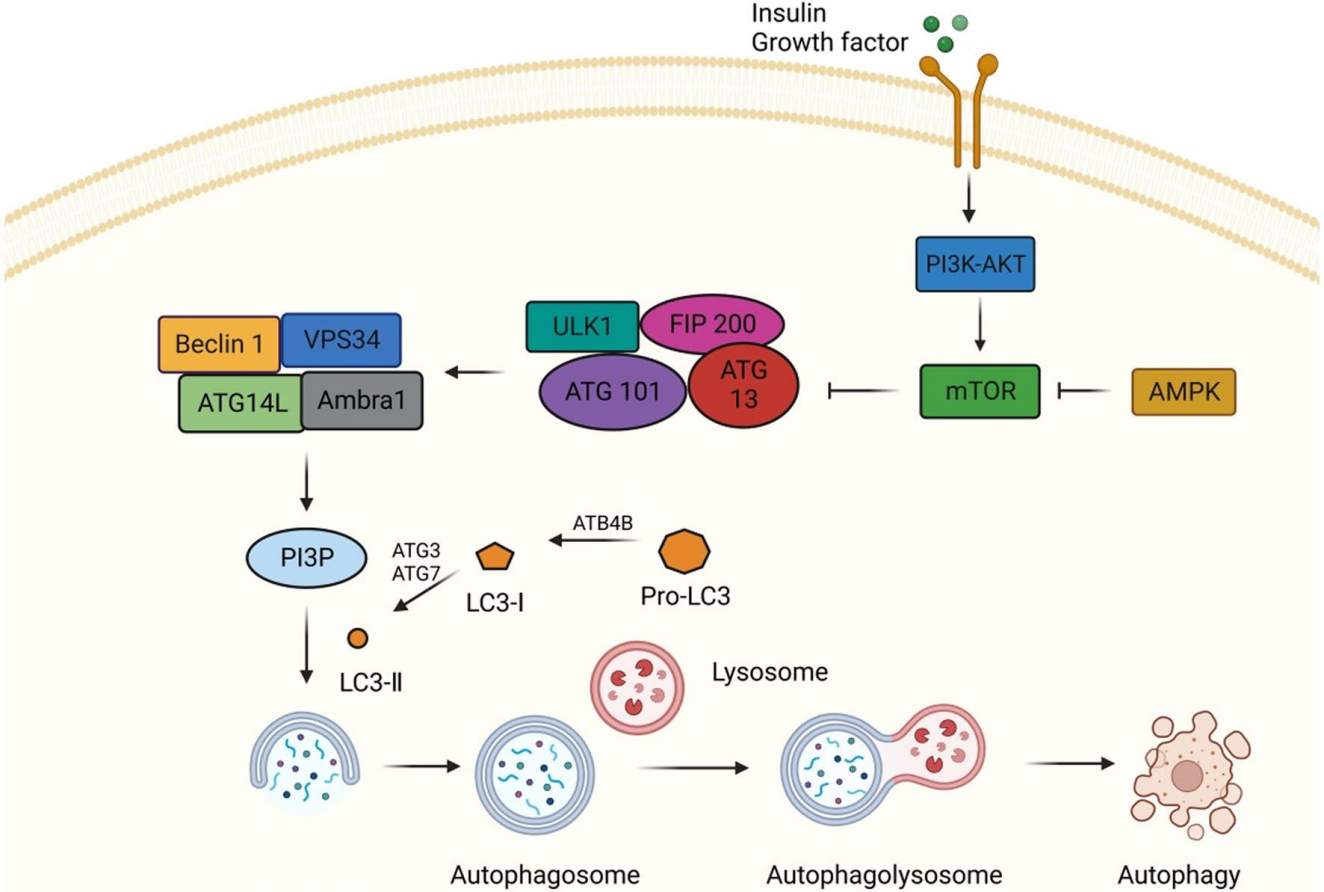
Autophagy-related pathway. Insulin or other growth factors can activate Class I phosphatidylinositol-3 kinase (PI3K)-AKT, thereby activating the mTOR pathway, and AMPK can negatively regulate the mTOR pathway. When activated, AMPK negatively regulates mTOR and activates the UNC-51-like kinase 1 (ULK1) complex, which includes ULK1, autophagy associated protein (ATG) 101, ATG13, and focal adhesion kinase interacting protein of 200 kDa (FIP200). Subsequently, ULK1 phosphorylates ATG14L, promoting the binding of Beclin1 to vacuolar protein sorter 34 (VPS34) to form the Beclin1 complex, which can promote the production of phosphatidylinositol-3-phosphate (PI3P), and thus promote the nucleation of autophagosome membrane. At the same time, the extension of autophagosomes also requires the participation of microtubule-associated protein 1 light chain 3 (LC3). The precursor form of LC3 is cleaved by the protease ATG4B to produce LC3-1. ATG7 and ATG3 are involved in the conversion of LC3-I (free form) to LC3-II (pe conjugated form). After the autophagosome is formed, it fuses with lysosomes to form autophagolysosomes, which eventually participate in autophagy

Autophagy, also known as cellular self-digestion, is a conserved catabolic process that degrades abnormal proteins, organelles, and macromolecules and recycles the decomposition products to maintain cell homeostasis and survival (Nishikawa et al. 2000; Klionsky and Emr 2000; Parzych and Klionsky 2014). The morphological characteristics of autophagy are enhanced substrate adhesion, focal expansion of the perinuclear space, expansion and fragmentation of the ER, early nuclear membrane curled, and late focal swelling of the perinuclear space. Autophagy is usually induced by nutrient deprivation, hypoxia, oxidative stress, genotoxic stress, or HG (Tang et al. 2019; Liu et al. 2022a, b). The most critical feature of autophagy is extensive cytoplasmic vacuolation to form autophagosomes, phagocytosis, and the subsequent lysosomal degradation (Glick et al. 2010). Autophagy is tightly regulated by a set of autophagy-related proteins encoded by a highly conserved set of genes (Fig. 3). The MAPK signaling pathway is also involved in the regulation of autophagy. For example, p38MAPK and JNK are involved in autophagy processes. Recent studies have reported that activation of JNK and p38α can induce autophagy (Yue and López 2020).

The autophagy activity of podocytes decreased after STZ induced diabetes, so autophagy may be involved in the pathogenesis of DKD. In general, podocytes have high basal autophagy levels, suggesting that autophagy is necessary for maintaining podocyte homeostasis (Bork et al. 2020). However, high blood glucose will reduce the level of autophagy, resulting in changes in podocyte function, and then damage the glomerular filtration barrier, and ultimately lead to the occurrence of DKD. For example, when podocytes are exposed to HG, they show reduced autophagy activity and levels of related proteins, including the Beclin-1 and Atg5-Atg12 complexes (Guo et al. 2017). Therefore, enhancing autophagy levels may be a potential way to treat DKD. LncRNA AK044604 (insulin sensitivity and autophagy regulator, Risa) and autophagy-related factors Sirt1 and GSK3β play important roles in DKD. Therefore, the down-regulation of Risa is considered an effective treatment to improve podocyte damage in DKD by modulating the Sirt1/GSK3β axis to enhance autophagy (Su et al. 2022). Mitochondrial dysfunction is a key mediator in the pathogenesis of DKD, and therapeutic strategies targeting mitochondrial dysfunction have considerable prospects. Zhou et al. found that progranulin (PGRN) maintains mitochondrial homeostasis through mitochondrial biogenesis and mitochondrial autophagy mediated by the PGRN-SIRT1-PGC-1α/FoxO1 signaling pathway, thereby preventing podocyte damage in DKD. This study provides an innovative therapeutic strategy for the treatment of DKD (Zhou et al. 2019; Fan et al. 2019).

When podocytes were exposed to HG, the mTORC1 pathway was activated and protective autophagy levels are reduced (Dai et al. 2017). Markus et al. demonstrated that the mTOR signaling pathway is of great significance in maintaining podocyte autophagy levels. Therefore, targeting mTOR-related signaling pathways to improve podocyte autophagy levels is expected to be a promising prospect for DKD therapy (Gödel et al. 2011). Currently, numerous studies have examined this topic. For example, Berberine (BBR), as an active component of Coptis, has various pharmacological effects, such as antioxidant, anti-inflammatory, and anti-diabetes effects, and it increases podocyte autophagy levels and reduces apoptosis by inhibiting mTOR/P70S6K/4EBP1 signaling pathway (Li et al. 2020). Similarly, mangiferin also protects podocytes by enhancing the AMPK-mTOR-ULK1 signaling pathway of autophagy, thus delaying the progression of DKD (Wang et al. 2018). In addition, the Jiedu Tongluo Baoshen formula (JTBF) also enhances DKD podocyte autophagy and reduces the production of proteinuria by inhibiting the PI3K/Akt/mTOR signaling pathway (Jin et al. 2022). More and more evidence suggests that actin cytoskeleton disturbance in podocyte injury is related to the PI3K signaling pathway. Huang et al. found that PI3K/Akt pathway is inactivated after podocyte injury, and Notoginsenoside R1 (NR1) treatment reactivates this pathway and further improves DKD (Huang et al. 2017). As multiple pathways are involved in the occurrence of autophagy, the ULK1 signaling pathway has attracted more and more attention. Geniposide improves DKD by enhancing ULK1-mediated autophagy in DKD mouse models, which indicates that geniposide is a promising treatment for DKD (Dusabimana et al. 2021). Therefore, maintaining the basal autophagy level of podocytes is essential for the effective treatment of DKD.

### Endoplasmic reticulum (ER) stress

**Fig. 4:**
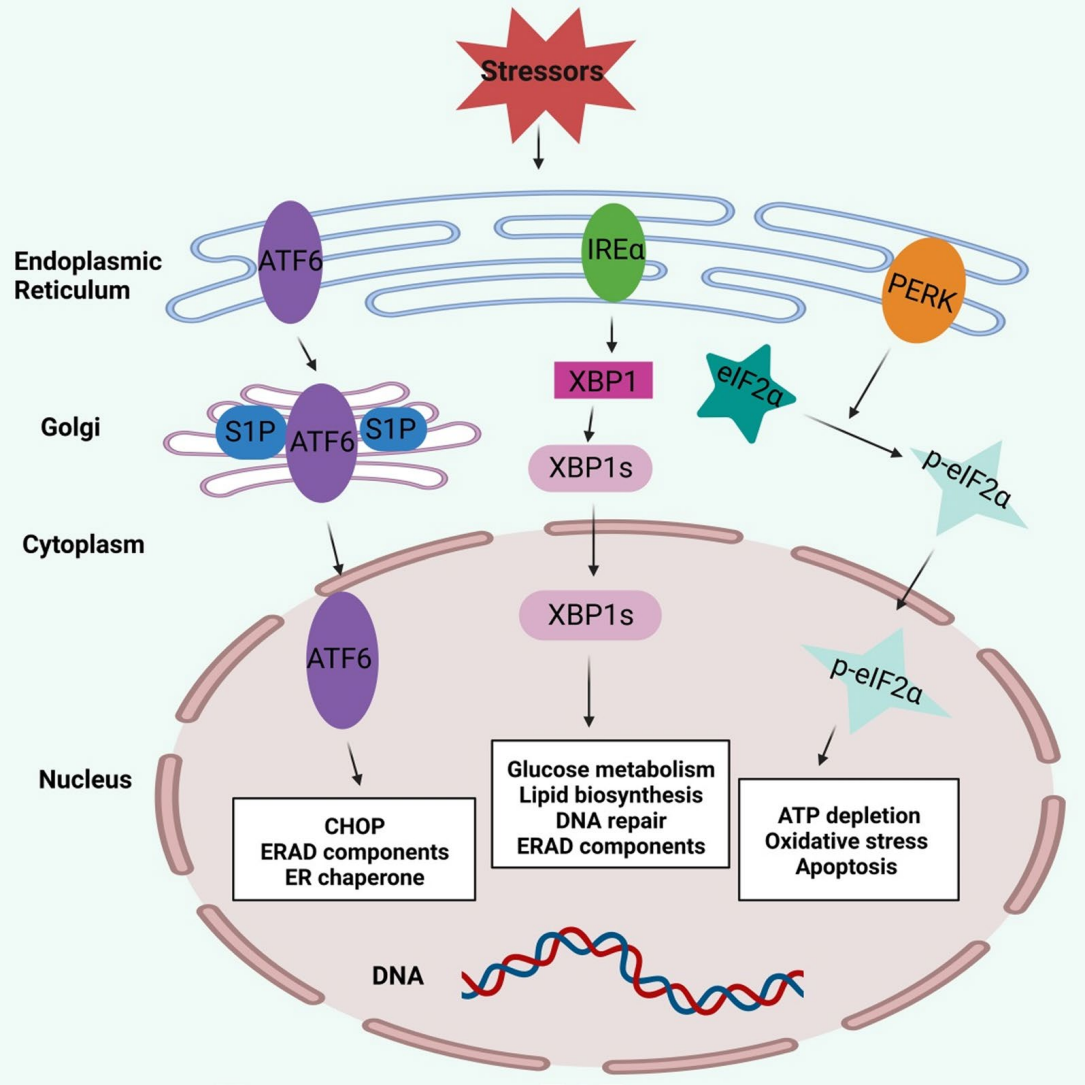
ER Stress-related pathway. ER stress mainly consists of three signaling pathways activated by three protein sensors, including activating transcription factor 6 (ATF6), inositol requirement enzyme 1α (IRE1α), and PRKR-like ER kinase (PERK). First, ATF6 moves to the Golgi apparatus, where it is sequentially cut by S1P and S2P. Subsequently activated ATF6 fragments mediate the expression of CHOP and several components of ER-associated degradation (ERAD). Second, IRE1α activation mediates the unconventional splicing of XBP1 mRNA. Spliced XBP1 (XBP1s) is involved in glucose metabolism, lipid biosynthesis, and DNA damage. Finally, activated PERK phosphorylates eIF2α, which is involved in ATP depletion, oxidative stress, and apoptosis

The ER is an organelle necessary for protein synthesis, folding and maturation in eukaryotic cells. Disruption of ER homeostasis leads to accumulation of unfolded or misfolded proteins, which in turn leads to ER stress and triggers the unfolded protein response (UPR) (Kaufman 2002). UPR mainly consists of three signaling pathways, which are activated by three protein sensors, including activating transcription factor 6 (ATF6), inositol requirement enzyme 1α (IRE1α), and PRKR-like ER kinase (PERK) (Fig. 4). In resting cells, these sensors bind glucose regulatory protein 78 (GRP78/BiP) in an inactive state. But when misfolded proteins accumulate in the ER, BiP separates from the sensor and binds to the unfolded protein, activating the sensor (Hetz 2012). Activation of UPR maintains ER function, promotes stress recovery, and has a protective effect against additional stress (adaptive UPR). In contrast, sustained or prolonged ER stress may be cytotoxic and eventually lead to cell death (Fig. 4) (Cunard and Sharma 2011).

Because the ER of podocytes has a high protein folding capacity and a high level of anabolic or catabolic activity, podocytes are very sensitive to ER stress. HG can induce ER stress of podocytes, which leads to the occurrence and development of DKD (Cao et al. 2014). Relevant studies have shown that compared with normal controls, DKD patients have up-regulated expression of related UPR genes, such as ER-companion GRP78 (Lindenmeyer et al. 2008). Upregulation of MicroRNA-27a (miR-27a) induces ER stress and damage in podocytes, leading to DKD. MicroRNAs can be regulated by changes in the activity of long non-coding RNAs (lncRNAs). Relevant experiments have shown that LINC01619, as a competing endogenous RNA (ceRNA), can regulate miR-27a/FOX01-mediated ER stress and podocellular injury in DKD (Bai et al. 2018).

HG can cause ER stress in podocytes, and related ER stress inhibitors can alleviate ER stress in podocytes. Fan et al. found that TUDCA, an ER stress inhibitor, improved diabetic kidney damage in a mouse model of progressive DKD. TUDCA treatment not only reduced proteinuria and renal histological changes in diabetic mice, but also improved podocyte and glomerular damage. The therapeutic mechanism may be related to the inhibition of ER stress markers in glomerular podocytes (Fan et al. 2017a, b). Meanwhile, Tian et al. found that emodin can improve kidney damage in DKD mice. Emodin mainly inhibits the upregulation of phosphorylated PERK, phosphorylated eIF2α, ATF4 and CHOP, and then inhibits HG-induced ER stress in podocytes, and finally improves DKD (Tian et al. 2018). Therefore, HG-induced ER stress and oxidative stress may be the cause of DKD. Oleanolic acid (OA) is found naturally in fruits and vegetables, and it has anti-inflammatory, lipid-lowering and antioxidant effects. N-acetylcysteine (NAC) is a precursor of glutathione and has a strong antioxidant effect in the body. Studies have found that OA and NAC can inhibit ER stress and antioxidant effects, so they have therapeutic effects on DKD (Lee et al. 2016). Finally, saturated free fatty acids can also induce ER stress, and researchers found that increasing dietary unsaturated free fatty acids can reduce ER stress and DKD related manifestations (Sieber et al. 2010). In recent years, the study of podocyte ER stress in DKD and its therapeutic agents have aroused great interest.

### Pyroptosis

**Fig. 5:**
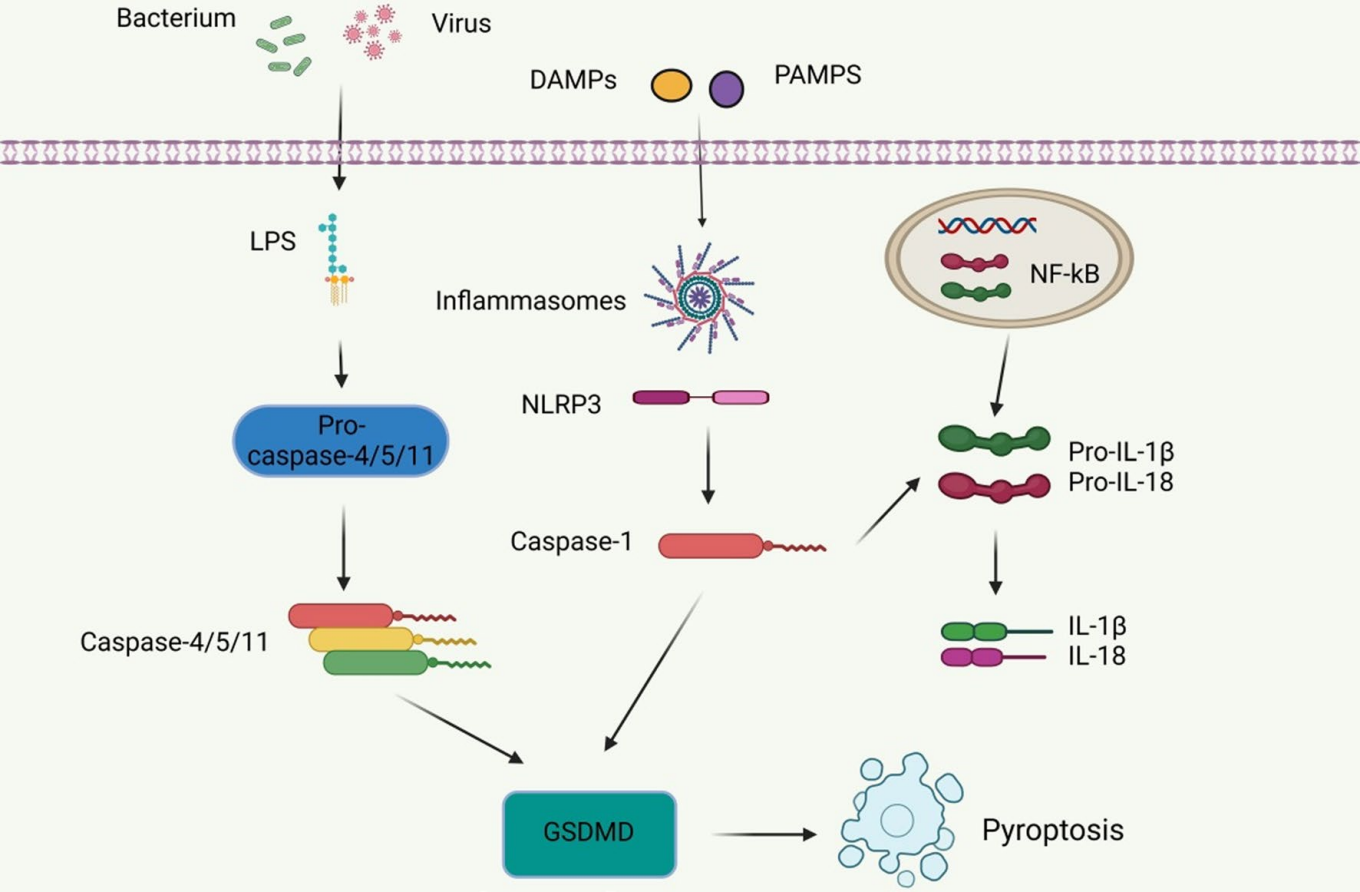
Pyroptosis-related pathway. Bacteria and viruses stimulate LPS, which in turn activates caspase-4/5/11, resulting in the formation of GSDMD, which is then involved in pyroptosis. In addition, harmful substances can also stimulate the formation of inflammasome and NLRP3, and subsequently activate caspase-1 to lead to the formation of GSDMD, thus triggering pyroptosis. In addition to the regulation of caspase-1, caspase-4/5/11 and GSDMD, pyroptosis is also regulated by several inflammatory mediators such as IL-1β and IL-18

Pyroptosis is a type of programmed cell death induced by the activation of caspase-1 by certain immunoreactive cells under the stimulation of pathogens and danger signals (Miao et al. 2010). Pyroptosis has the morphological characteristics of both apoptosis and necrosis, including nuclear contraction, DNA breakage, positive staining, cell swelling and rupture, and an inflammatory reaction (Cao et al. 2022). Pyroptosis is characterized by pore formation, cell lysis, the release of pro-inflammatory cytokines and cell contents, and the activation of the inflammasome. The inflammasome is a molecular platform that causes caspase-1 activation and interleukin-1β and IL-18 secretion during cell infection or stress (Fantuzzi and Dinarello 1999). Activation of the nucleotide-bound oligomeric domain-like receptor protein 3 (NLRP3) inflammasome, a key component of pyroptosis, induces Caspase-1 activation. Activated caspase-1 cleaves gasdermin D (GSDMD) to generate an n-terminal GSDMD fragment, leading to the formation of membrane pores and the subsequent inflammatory responses (Fig. 5) (Lee et al. 2019; Zhaolin et al. 2019).

It has been reported that pyroptosis is related to the loss of podocytes (Haque et al. 2016). Podocyte pyroptosis has been observed in both db/db mouse models and STZ-treated mice, and podocytes are one of the promoters of IL-1β under many pathological conditions (Niemir et al. 1997). NADPH oxidase in podocytes mainly leads to the upregulation of the activated NLRP3 inflammasome, the recruitment of a large number of immune cells, and ultimately to glomerular injury (Gao et al. 2015; Wu et al. 2021). NLRP3, as a key component of pyroptosis, has attracted increasing attention, and targeting NLRP3 activation and formation has great potential in the treatment of DKD. For example, gene therapy involving IL-22 plays a large role in inhibiting the activation of the NLRP3 inflammasome during podocyte pyroptosis, thereby reducing renal fibrosis and DKD progression (Wang et al. 2017). The inhibition of MiR-21-5p in macrophage-derived extracellular vesicles and the subsequent regulation of A20 reduces the levels of pyroptosis-related inflammasome NLRP3, caspase-1, and IL-1β, reducing the production of ROS and alleviating podocyte injury (Ding et al. 2021). Meanwhile, Cheng et al. found that Caspase-11/4 and GSDMD-dependent podocyte pyroptosis were related to the development of DKD, and both caspase-11 and GSDMD knockout mice significantly improved the deterioration of renal function and the morphological changes in the glomerulus and podocyte (Cheng et al. 2021).

In addition, there are currently several drug therapies that target NLRP3. For example, solasonine (SS) alleviates HG-induced podocyte pyroptosis and oxidative damage by modulating the Nrf2/NLRP3 signaling pathway (Zhang et al. 2022). The total flavones of Abelmoschus manihot (TFA) (the medicinal parts are the corolla with stamens and style) improve podocyte pyroptosis and injury in HG by targeting N6-methyladenosine (m^6^A) modification-mediated NLRP3 inflammasome activation and the PTEN/PI3K/Akt signaling pathway (Liu et al. 2021; Zhang et al. 2014; Lu et al. 2021). Moreover, Fucoidan (FPS) inhibits NLRP3 inflammasome-mediated podocyte pyroptosis by modulating the AMPK/mTORC3/NLRP1 signaling axis in DKD, thereby alleviating DKD (Wang et al. 2022a, b, c). These findings provide insight into targeted therapies for DKD. In addition, carnosine, a dipeptide composed of β-alanine and L-histidine, has shown great potential in targeting caspase-1 to inhibit DKD podocyte pyroptosis. MPC5 cells cultured in HG and STZ-induced diabetic mouse models were used. Zhu et al. found that carnosine significantly reversed albuminuria and histopathological changes in STZ-induced diabetic mice, and alleviated kidney inflammation and pyroptosis (Zhu et al. 2021). These findings confirm the unique role of pyroptosis in DKD and suggest that inhibition of the pyroptosis signaling pathway can expand the potential therapeutic targets for DKD treatment.

### Necroptosis

**Fig. 6:**
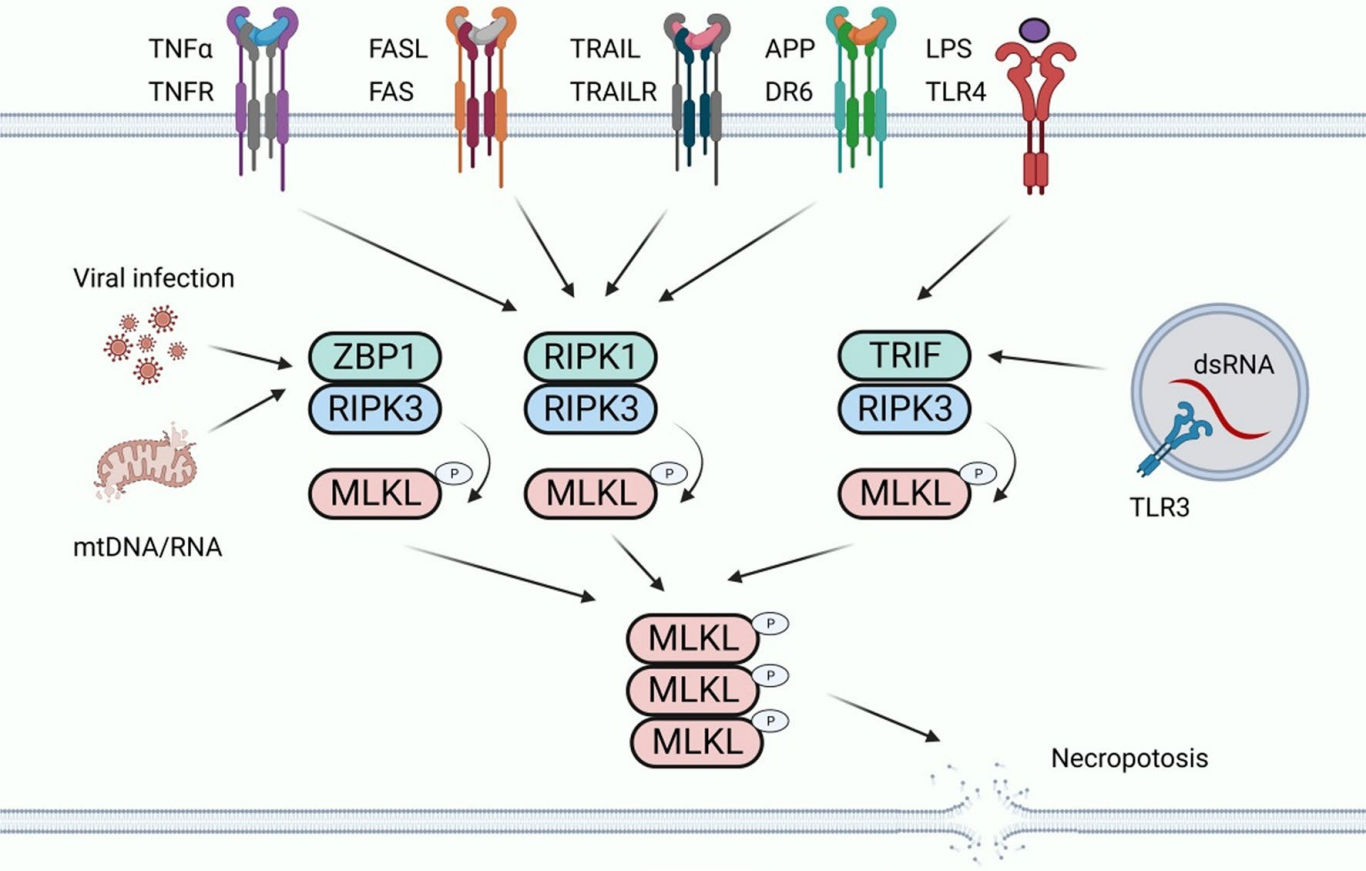
Necroptosis-related pathway. Necroptosis can be engaged by the ligation of TNF receptor family proteins (including TNFR, FAS, TRAILR, and DR6) through RIPK1–RIPK3 when Caspase-8 activity is blocked. Necroptosis can be also triggered by the activation of TLR3 and TLR4 by double stranded RNA (dsRNA) and LPS in macrophages, respectively, through TRIF-dependent activation of RIPK3. Viral RNA and the released DNA/RNA from damaged mitochondria can induce necroptosis by ZBP1-dependent activation of RIPK3. Activated RIPK3 phosphorylates MLKL and leads to the subsequent oligomerization of MLKL. The oligomerized MLKL translocates to the plasma membrane and engages ion channels and mediates plasma membrane rupture

Necroptosis is another type of programmed death that has the characteristics of both apoptosis and necrosis (Tang et al. 2019). Its morphological features are similar to necrosis, with organelle swelling and nuclear membrane fragmentation (Liu et al. 2018). In addition, the integrity of the plasma membrane is compromised, leading to rupture of the plasma membrane and cell content leakage, which ultimately leads to inflammation (Dhuriya and Sharma 2018). Necroptosis is triggered by interleukin-1β (IL-1β), TNF, certain viral infections, and other factors (Yu et al. 2021). When necroptosis occurs, cell contents are released through the ruptured plasma membrane, driven by a signaling cascade of receptor-interacting protein kinase 1 (RIPK1), receptor-interacting protein kinase 3 (RIPK3), and mixed lineage kinase-like domains (MLKL) to activate the inflammatory response (Fig. 6) (Zhang et al. 2018; Yoshida 2017; Grootjans et al. 2017). In the context of HG, the necrosis activation pathway is mediated by death receptor ligands, such as tumor necrosis factor receptor 1 (TNFR1) and Fas receptor. Tnfr1-mediated necrosis is the most thoroughly studied pathway. After TNF-α binds to TNFR1, different signaling complexes, namely, pro-survival complex I, pro-apoptotic complex IIa, and necrotic complex IIb, initiate various functions, namely cell survival, apoptosis, or necrosis (Galluzzi et al. 2018).

Necroptosis plays an important role in podocyte injury, so it may be involved in the pathogenesis of DKD (Sosna et al. 2013). Studies have shown that podocyte necroptosis is closely related to ubiquitin C-terminal hydrolase L1 (UCHL1), which regulates the ubiquitination state of RIPK1/RIPK3 pathway. Abnormal overexpression of UCHL1 in podocytes leads to dysubiquitination of RIPK1/RIPK3 pathway, which stimulates necroptosis and injury of podocytes, and ultimately produces DKD (Erekat 2022a, b). Xu et al. found that UCHL1, as a member of the deubiquitination enzyme group, was overexpressed in the podocytes of DKD patients. Under DKD conditions, HG stimulation induces podocyte necroptosis by activating RIPK1 and RIPK3 pathways, which is accompanied by increased UCHL1 expression. Incremental UCHL1 further enhances the activation of the RIPK3/MLKL pathway and promotes podocyte necroptosis. Therefore, UCHL1 promotes HG-induced podocyte necroptosis by regulating the ubiquitination state of the RIPK1/RIPK3 pathway. The above studies demonstrate that the RIPK1/RIPK3 pathway provides a new idea for targeting DKD podocyte necroptosis. For example, deletion of the UCHL1 gene shortens the half-life of RIPK1 and RIPK3 proteins and thus reduces their expression (Xu et al. 2019).

Meanwhile, necrostatin 1 (Nec1) reduces DKD podocyte necroptosis and the subsequent damage by decreasing the expression levels of RIPK1 and RIPK3 (Xu et al. 2019).In addition, paeoniflorin (PF) directly binds and promotes the degradation of TNFR1 in podocytes in an STZ-induced mouse diabetes model and an HG-induced podocyte injury model. It regulates the RIPK1/RIPK3 signaling pathway to affect necroptosis, thereby preventing DKD podocyte injury (Wang et al. 2022a, b, c). Moreover, curcumin therapy prevents HG-induced podocyte necroptosis by inhibiting ROS production and the abnormal expression of RIPK3 (Chung et al. 2022). These findings demonstrate that necroptosis is a viable cellular target for DKD treatment.

### Ferroptosis

**Fig. 7:**
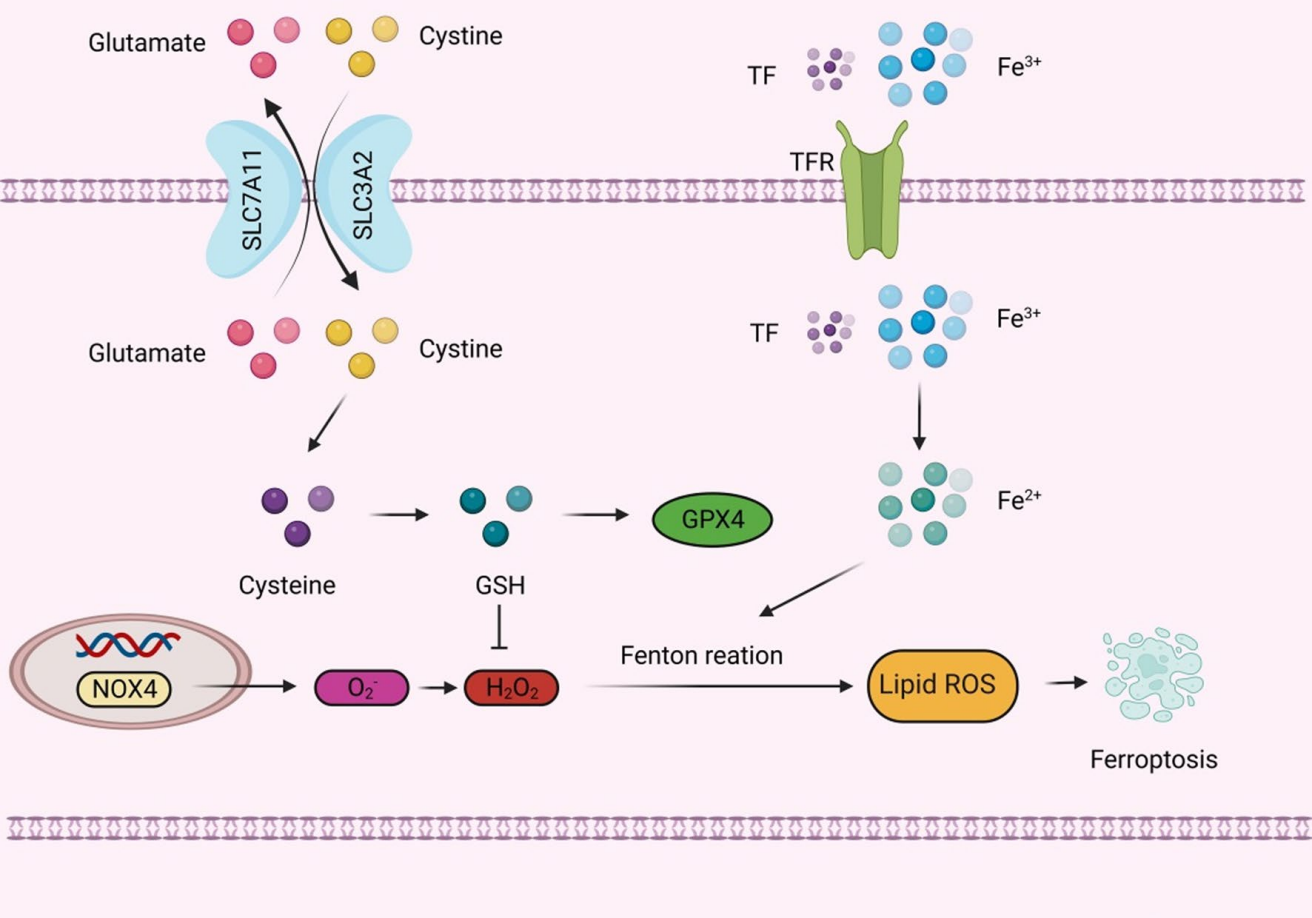
Ferroptosis-related pathway. Glutamate inhibits cystine uptake by the cystine-glutamate antitransporter (system Xc^−^), which subsequently leads to glutathione (GSH) depletion and the inactivation of the phospholipid peroxidase glutathione peroxidase 4 (GPX4), which promotes the accumulation of H_2_O_2_, and NOX4, which also promotes the accumulation of H_2_O_2_. Both eventually lead to ferroptosis. In addition, dysfunction of iron metabolism in cells can also lead to ferroptosis. The intracellular iron level is mainly regulated by transferrin receptor (TFR), and the increase of TFR expression will cause more Fe^3+^ to enter the cell, and Fe^3+^ will be reduced to Fe^2+^ by iron reductase, and the production of ROS will be promoted through Fenton reaction, which ultimately leads to ferroptosis

Dixon first proposed the concept of ferroptosis in 2012, which is an iron-dependent, non-apoptotic mode of cell death characterized by the accumulation of lipid ROS (Dixon et al. 2012). Ferroptosis occurs primarily in cells and is characterized by a reduction in mitochondrial volume, an increase in the density of the bilayer membrane, and a reduction or disappearance of the mitochondrial crest, but the cell membrane remains intact, the nucleus is normal in size, and chromatin is not concentrated (Dixon et al. 2012; Yang and Stockwell 2008). When intracellular glutathione (GSH) is depleted and glutathione peroxidase 4 (GPX4) activity is reduced, lipid peroxides cannot be metabolized through a GPX4-catalyzed reduction reaction, and Fe^2+^ oxidizes lipids in a Fenton and Haber Weis-like manner, producing large amounts of ROS and promoting ferroptosis (Fig. 7) (Yang and Stockwell 2008; Friedmann Angeli et al. 2014). Ferroptosis can be activated by degenerative processes or induced by anticancer therapy (Dixon et al. 2012).

Related studies have found that ferroptosis is involved in kidney injury in STZ-induced type I diabetic mice and db/db mice, and the expression levels of SLC7A11 and GPX4 are significantly down-regulated in kidney biopsy samples from diabetic patients. The related mechanism may be that HG induces the production of ROS and MDA in podocytes, and inhibits the synthesis and accumulation of SOD and GSH. Due to the high sensitivity of podocytes to ROS, excessive ROS can cause irreversible changes in the structure and function of podocytes, leading to ferroptosis and the occurrence of DKD. Therefore, there is increasing evidence that ferroptosis promotes the development of DKD, and inhibiting ferroptosis may be a way to treat DKD (Wang et al. 2023; Xiong et al. 2023). The process of ferroptosis is accompanied by the excessive production of lipid ROS, which can lead to oxidative stress. Kidney cells are rich in mitochondria and thus are more vulnerable to oxidative stress damage. Oxidative stress is part of the pathogenesis of DKD, which indicates that ferroptosis may be related to DKD, and oxidative stress is expected to be one of the potential targets for the treatment of ferroptosis in DKD podocytes. For example, the upregulation of peroxidoreductin 6 (Prdx6) prevents podocyte damage in DKD by alleviating oxidative stress and ferroptosis (Xiong et al. 2023).

Also, the ACSL4 inhibitor rosiglitazone mitigates kidney pathological damage in DKD mice by reducing lipid peroxidation (Xiong et al. 2023). In addition, ginkgolide B (GB), the active ingredient of ginkgo biloba extract, effectively reduces total cholesterol, triglyceride concentrations, and lipid accumulation in podocytes by in vivo and in vitro experiments, and its mechanism mainly involves reducing oxidative stress and ferroptosis by inhibiting GPX4 ubiquitination, thereby improving DKD (Chen et al. 2022). Oxidative stress is controlled by multiple pathways and is associated with ferroptosis-related regulators. Mangiferin monosodium salt (MGM) up-regulates the mevalonate-mediated antioxidant capacity (GPX4 and ferroptosis suppressor 1/CoQ10 axis) and impairs the production of ACSL4-mediated lipid drivers in the kidney. It improves renal ferroptosis in DKD rats induced by STZ (Zhao et al. 2023).

### Mitotic catastrophe

**Fig. 8:**
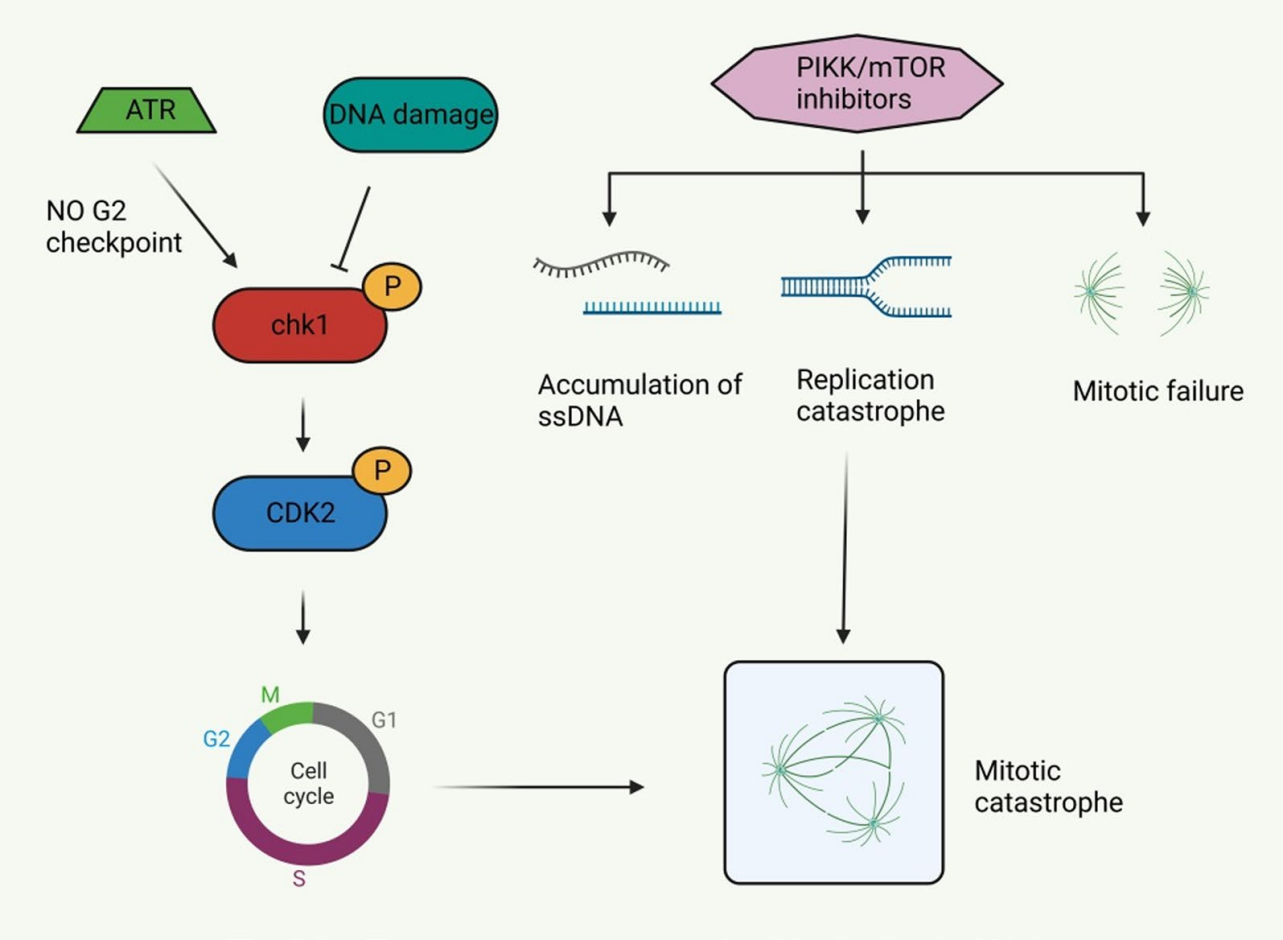
Mitotic catastrophe-related pathway. The rad3-associated protein (ATR)-chk1 signaling pathway is activated in the absence of G2 checkpoints, and restoring S/G2 and G2/M cell cycle checkpoints can avoid mitosis disasters. DNA damage inhibits checkpoint kinase 1 (chk1) and cyclin-dependent kinase (CDK) 2, which in turn inhibits the recovery of cell cycle checkpoints and ultimately leads to mitotic catastrophe. In addition, PI3K-like kinase (PIKK)/mTOR inhibitors cause single-stranded deoxyribonucleic acid (ssDNA) accumulation, replication mutations, and mitotic failure, and eventually mitotic catastrophe as well

Mitotic catastrophe (MC) is a delayed mitosis-related cell death mechanism resulting from the premature or inappropriate entry of cells into mitosis and the loss of cell cycle checkpoints. Morphological features include multiple centrosomes, chromosomal dislocation, abnormal mitotic spindle, micronucleus, or irregularly shaped nucleus. MC is usually triggered by chemical or physical stress (Castedo et al. 2004; Vitale et al. 2011). A variety of molecules are involved in the regulation of MC, especially cell cycle-specific kinases (such as cyclin B1-dependent kinases CDK1 and aurora kinases), cell cycle checkpoint proteins, survivin, p53, caspases, and the Bcl-2 family (Fig. 8) (Castedo et al. 2004).

Related studies found mitotic podocytes in the podocytes of DKD patients, which were characterized by binucleation, chromatin concentration, and loss of foot process, suggesting the possibility of MC in podocytes (Liapis et al. 2013). Mature podocytes are considered to be G0 phase quiescent cells that lack the ability to proliferate (Liapis et al. 2013). Although podocytes cannot divide after damage, they can re-enter the cell cycle (Hagen et al. 2016). However, due to the lack of auroral kinase B expression, mature podiocytes cannot form effective mitotic spindles (Lasagni et al. 2010, 2013). Therefore, differentiated podocytes are inherently resistant to mitosis, and their proliferative response does not promote damage recovery, but instead accelerates cell loss, leading to MC and eventually DKD in podocytes (Liapis et al. 2013).

Currently, there are few relevant targeted therapy studies on MC in DKD podocytes, and there is a lack of relevant effective data and clinical trials, but its important role in DKD has become increasingly impossible to ignore. For example, Wang et al. found that long non-coding RNA (lncRNA) MIAT was significantly upregulated in the plasma and kidney tissue of patients with DKD, and inhibition of lncRNA MIAT prevented podocyte injury and MC in DKD (Wang et al. 2022a, b, c). Myeloid-derived growth factor (MYDGF) promotes the production of glucagon-like peptide-1 and improves glucose/lipid metabolism in diabetic mouse models. MYDGF alleviates podocyte injury and proteinuria by activating the Akt/BAD signaling pathway in STZ-induced diabetic mice and HG-cultured podocyte models. MYDGF deficiency, on the other hand, exacerbates MC in podocytes in DKD, suggesting that MYDGF may be an attractive therapeutic target for DKD (He et al. 2020; Zhan et al. 2022). As a new model of podocyte death, MC is expected to be the focus of future research.

### Other types of podocytes death in DKD

Anoikis is a cell defect caused by loss of attachment or improper adhesion of extracellular matrix. The foot process connects the podocytes to GBM through integrins and dystroglycans. When anoikis occurs, the foot process is completely detached from GBM, resulting in the disappearance of podocytes (Reddy et al. 2008). In DKD patients and STZ-induced diabetic rats, the expression of α3β1 integrin is reduced, resulting in focal detachment of GBM from podocytes, suggesting that anoikis is involved in the pathogenesis of DKD (Sawada et al. 2016).

Podoptosis is a type of cell death associated with over-activation of p53. Maintaining the balance of p53 is known to be essential for the survival of podocytes, which are rich in many proteins that interact with the p53 pathway, such as WT-1, MDM2, and RARRES1. MDM2 could promote podocyte loss by overriding cell cycle G2/M restriction and entering mitosis through degradation of p53 or by retaining p53 in the cytosol (Thomasova et al. 2015). Morphologically, podoptosis is characterized by massive cytoplasmic vacuolization and signs of ER stress (Yin et al. 2021). However, there is little evidence of a link between podoptosis and DKD. With the progress of research, more associations between the two may be found in the future, providing new ideas for the treatment of DKD.

## Summary and outlook

**Fig. 9:**
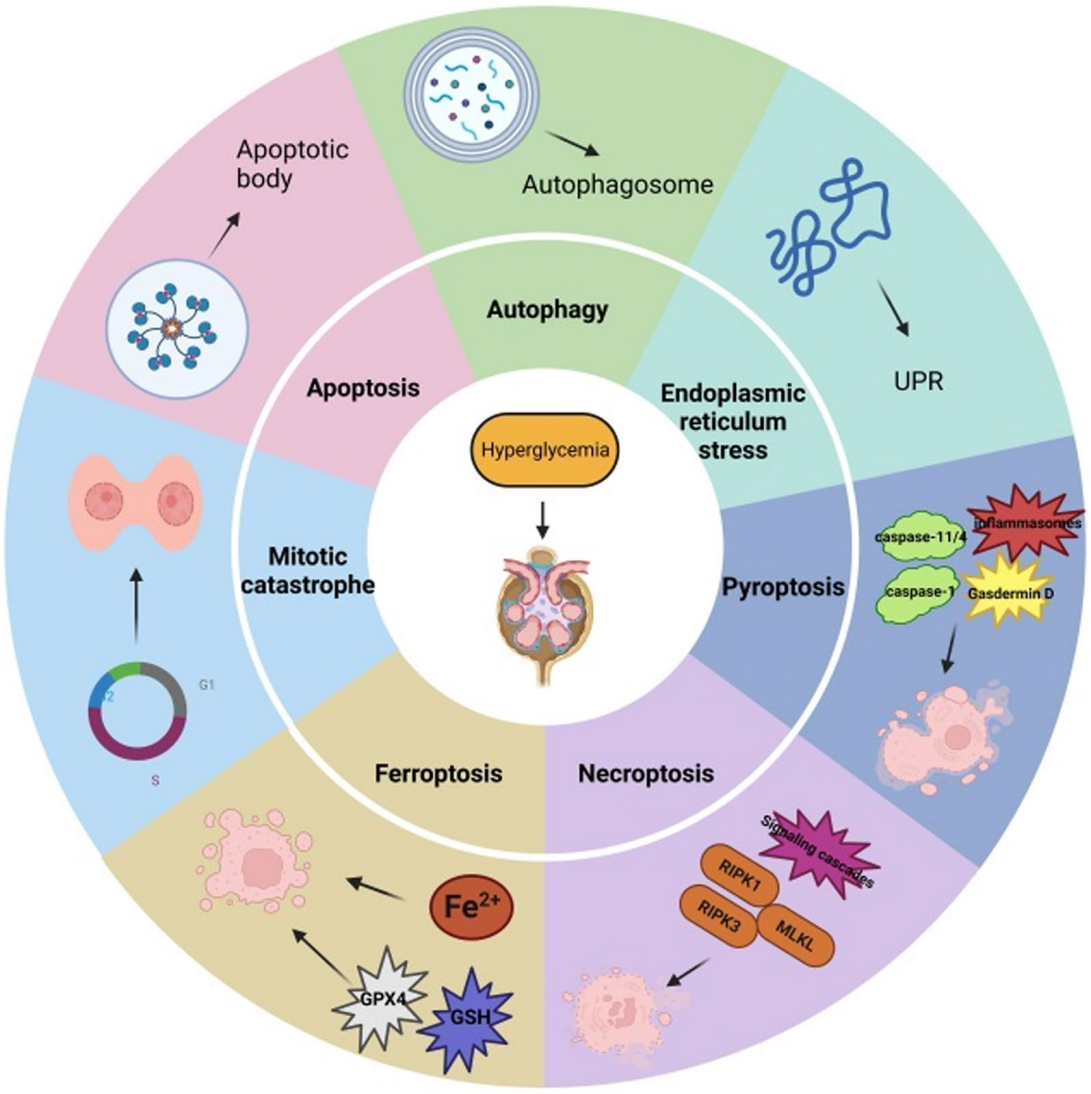
Modes of podocyte death in DKD. Apoptosis is characterized by nuclear condensation and the formation of apoptotic bodies. Autophagy is characterized by extensive cytoplasmic vacuolization leading to the formation of an autophagosome, phagocytosis, and subsequent lysosomal degradation. ER stress is caused by excessively prolonged unfolded protein response (UPR) UPR. Pyroptosis activates inflammatory factors to aggravate podocyte injuries. Necroptosis drives signaling cascades, such as receptor-interacting protein kinases 1 and 3 and mixed lineage kinase domain-like, ultimately promoting the death of podocytes. Ferroptosis is an iron- and lipotoxicity-dependent form of regulated cell death (RCD), and MC mediates a faulty mitotic process

DKD is the most important microvascular complication of diabetes mellitus, and its occurrence and development are closely related to the injury and loss of podocytes. Various cell death modes are involved in the occurrence and development of DKD (Fig. 9). This paper specifically reviewed the types and mechanisms of podocyte death in DKD, as well as the main targeted therapies and drugs (Table 2). However, the specific pathophysiological mechanisms related to the cell death modes in podocytes are still not completely clear, and relevant studies and available data are few. Thus, the relationship between various forms of cell death and DKD require further exploration, as many questions remain unanswered. We know that apoptosis is the most important form of cell death, which is mainly involved in the death of podocytes in DKD through exogenous and endogenous pathways. In addition, autophagy generally plays a beneficial role in cells and in some cases mediates podocyte damage. However, studies on podocyte autophagy in DKD remain scarce, and direct evidence of the role of autophagy in the development of DKD is still lacking. ER stress is caused by excessively prolonged unfolded protein response (UPR) UPR. Pyroptosis has been identified as a unique mode of cell death and is closely related to the activation of the inflammasome. However, how it promotes the development of DKD remains to be clarified. In addition, necroptosis is driven by a cascade of signals, such as RIPK1, RIPK3, and MLKL. Moreover, during ferroptosis ROS accumulation causes cellular damage due to oxidative stress, and finally, mitotic catastrophe causes abnormal podocyte division and accelerates their loss. However, there are few animal models on necroptosis, ferroptosis, and mitotic catastrophe in podocytes related to DKD. Thus, more data is needed to draw strong meaningful conclusions.

**Table 2:** Potential approaches to target podocyte death in DKD

Mode of cell death	Treatment	Mechanism of action	DKD model	References
Apoptosis	TXNIP gene was deleted	Termination of O^2−^ production in mitochondria and inhibition of oxidative stress	STZ-induced TxNIP WT (TxNIP), TxNIP KO (TxNIP^+/+−/−^), TxNIP HET (TxNIP^+/−^), and Hcb-19 mice Hg-induced conditionally immortalized human podocytes	Shah et al. (2015)
	Sestrin2 was overexpressed	Inhibition of the TSP-1/TGF-β1/Smad3 pathway inhibits oxidative stress	STZ-induced C57BL/6J mice Hg-induced MPC5 cells	Shan et al. (2015)
	ADSC-Exo	The Smad1/mTOR pathway was inhibited by enhancing miR-486 expression	C0010BL/KsJ db/db mice Hg-induced MPC5 cells	Jin et al. (2019)
	Telmisartan	Inhibition of swiprosin-1 expression further inhibited the p38 MAPK pathway	STZ-induced Sprague–Dawley rats Hg-induced MPC5 cells	Wei et al. (2022)
	Wogonin	Enhanced anti-apoptotic Bcl-2 activation	STZ-induced C57BL/6J mice Hg-induced MPC5 cells	Liu et al. (2022a, b)
	AS-IV	Activation of PPARγ-Klotho-FoxO1 pathway inhibits oxidative stress	C57BLKS/J-LepR db/db mice Hg-induced MPC5 cells	Xing et al. (2021)
Autophagy	Down-regulation of Risa	Inhibition of the Sirt1/GSK3β pathway	C57BL/KsJ db/db mice Hg-induced MPC5 cells	Su et al. (2022)
	PGRN	Enhanced PGRN-Sirt1-PGC-1α/FoxO1 pathway	STZ-induced C57BL/6 mice	Zhou et al. (2019)
	BBR	Inhibition of the mTOR/P70S6K/4EBP1 pathway	Hg-induced MPC5 cells	Li et al. (2020)
	Mangiferin	It promoted the up-regulation of AMPK phosphorylation, the down-regulation of mTOR phosphorylation, and the up-regulation of p-ULK1	STZ-induced Sprague–Dawley rats Hg-induced MPC5 cells	Wang et al. (2018)
	JTBF	Inhibition of the PI3K/Akt/mTOR pathway	STZ-induced Sprague–Dawley rats	Jin et al. (2022)
	NR1	Activation of the PI3K/Akt pathway	Hg-induced conditionally immortalized human podocytes	Huang et al. (2017)
	Geniposide	Enhanced AMPK activity	STZ-induced C57BL/6 mice	Dusabimana et al. (2021)
ER stress	LINC01619	Inhibition of the miR-27a/FOXO1 pathway	STZ-induced Sprague–Dawley rats Hg-induced MPC5 cells	Bai et al. (2018)
	TUDCA	It inhibits the expression of ER stress markers	C57BL/KsJ db/db mice Hg-induced conditionally immortalized human podocytes	Fan et al. (2017a, b)
	Emodin	Inhibition of the PERK-eIF2α pathway	STZ-induced C57BL/6J mice Hg-induced MPC5 cells	Tian et al. (2018)
	OA	Enhance antioxidant effect	C57BL/KsJ db/db mice	Lee et al. (2016)
	NAC	Enhance antioxidant effect	C57BL/KsJ db/db mice	Lee et al. (2016)
	Unsaturated free fatty acids	Attenuate the upregulation of CHOP	Hg-induced MPC5 cells	Sieber et al. (2010)
Pyroptosis	Gene therapy for IL-22	Inhibition of NLRP3 inflammasome activation	STZ-induced C57BL/6 mice	Wang et al. (2017)
	Inhibition of MiR-21-5p in macrophage-derived EVs	It also reduced the levels of NLRP3, caspase-1, and IL-1β, which are inflammasomes associated with pyroptosis	STZ-induced C57BL/6 mice Hg-induced MPC5 cells	Ding et al. (2021)
	Knockdown of caspase-11 or GSDMD	Inhibition of caspase-11 and GSDMD-mediated pyroptosis	STZ-induced C57BL/6J mice Hg-induced MPC5 cells	Cheng et al. (2021)
	SS	Inhibition of the Nrf2/NLRP3 pathway	Hg-induced MPC5 cells	Zhang et al. (2022)
	TFA	Targeting m^6^A modifation-mediated NLRP3 inflammasome activation and the PTEN/PI3K/Akt pathway	Hg-induced MPC5 cells	Liu et al. (2021)
	FPS	Regulating the AMPK/mTORC3/NLRP1 pathway inhibits the activation of NLRP3 inflammasome	STZ-induced Sprague–Dawley rats Hg-induced MPC5 cells	Wang et al. (2022a, b, c)
	Carnosine	Targeting caspase-1	STZ-induced C57BL/6J mice Hg-induced MPC5 cells	Zhu et al. (2021)
Necroptosis	The UCHL1 gene was deleted	This results in a shortened half-life of RIPK1 and RIPK3 proteins and a decrease in their expression	Hg-induced MPC5 cells	Xu et al. (2019)
	Nec1	The expression of RIPK1 and RIPK3 was inhibited	Hg-induced MPC5 cells	Xu et al. (2019)
	PF	It directly binds and promotes the degradation of TNFR1 in podocytes, thereby inhibiting the RIPK1/RIPK3 pathway	STZ-induced C57BL/6J mice Hg-induced MPC5 cells	Wang et al. (2022a, b, c)
	Curcumin	Inhibition of ROS production and abnormal expression of RIPK3	Hg-induced MPC5 cells	Chung et al. (2022)
Ferroptosis	The expression of Prdx6 was up-regulated	Alleviates oxidative stress	STZ-induced C57BL/6 mice Hg-induced MPC5 cells	Zhang et al. (2022)
	Rosiglitazone	Inhibition of ACSL4 expression reduces lipid peroxidation	STZ-induced ICR mice C57BL/KsJ db/db mice	Wang et al. (2021)
	GB	Inhibition of GPX4 ubiquitination	C57BL/KsJ db/db mice Hg-induced MPC5 cells	Chen et al. (2022)
	MGM	Up-regulation of mevalonate-mediated antioxidant capacity and impairment of ACSL4-mediated lipid driver production in the kidney	STZ-induced Sprague–Dawley rats	Zhao et al. (2023)
Mitotic catastrophe	Inhibition of lncRNA MIAT	It increases the expression of Sox4, which in turn regulates p53 ubiquitination and acetylation	STZ-induced C57BL/6 mice	Wang et al. (2022a, b, c)
	MYDGF	Activation of the Akt/BAD pathway	STZ-induced C57BL/6J mice Hg-induced MPC5 cells	He et al. (2020)

Importantly, this review suggests that any type of cell death is a promising therapeutic target that may, in the future, correct poor outcomes in podocytes and the development of DKD. In addition, understanding whether different types of cell death interfere with each other during DKD is crucial for the precise treatment of the disease. The various forms of cell death are likely to overlap at the same stage but have varying contributions to DKD at different stages. This is another direction worth studying in the future. For example, p53 is involved in a variety of cell death processes. Under cell stress, p53 promotes endogenous apoptosis by activating the expression of pro-apoptotic genes, such as BAX, APAF-1, PUMA, NOXA, and p53AIP13, or by inhibiting the expressions of the anti-apoptotic genes BCL-2 and BCL-xL (Speidel 2010; Bieging et al. 2014). At the same time, p53 has a dual effect on autophagy, because it can induce or inhibit autophagy, depending on its location in the cell (Maiuri et al. 2010). Many genes and proteins are involved in the regulation of ferroptosis, including GPX4, SLC7A11, and p53 (Dixon et al. 2012). In addition, p53 is also associated with a variety of cell death modes, such as pyroptosis and mitotic catastrophe (Ranjan and Iwakuma 2016). Diabetes is considered an oxidative stress and a chronic inflammatory disease. ROS is regarded as an important pathogenesis of DKD. ROS also plays an important role to activate programmed cell death pathways, including apoptosis, autophagy and Ferroptosis (Jha et al. 2016). Regulated cell death includes pyroptosis, necroptosis and ferroptosis can trigger a strong inflammatory immune response (Wei and Szeto 2019; Li et al. 2023). Clearly, our search for DKD podocellular death is still in its early stages, and our understanding of this issue is far from comprehensive and in-depth. Therefore, future directions in this area of research include elucidating the specific pathophysiological mechanisms of the various cell death types in the occurrence and development of DKD and whether the different types of cell death interfere with each other during DKD.

## Data Availability

Not applicable.
